# Discovery of Novel Inhibitors Targeting Multi-UDP-hexose Pyrophosphorylases as Anticancer Agents

**DOI:** 10.3390/molecules25030645

**Published:** 2020-02-03

**Authors:** Yueqin Yang, Hariprasad Vankayalapati, Manshu Tang, Yingbo Zheng, Yingri Li, Cong Ma, Kent Lai

**Affiliations:** 1 Department of Pediatrics, University of Utah School of Medicine, Salt Lake City, UT 84108, USA; Yueqin.Yang@hsc.utah.edu (Y.Y.); manshu.tang@hsc.utah.edu (M.T.); annie826.li@gmail.com (Y.L.); 2 Department of Medicinal Chemistry, University of Utah School of Pharmacy, Salt Lake City, UT 84108, USA; hari@hci.utah.edu; 3 Huntsman Cancer Institute, University of Utah, Salt Lake City, UT 84108, USA; 4 State Key Laboratory of Chemical Biology and Drug Discovery, Department of Applied Biology and Chemical Technology, Hong Kong Polytechnic University, Kowloon, Hong Kong; jason.zheng@polyu.edu.hk

**Keywords:** galactose-1 phosphate uridylyltransferase (GALT), UDP-glucose pyrophosphorylase (UGP2), UDP-*N*-acetylglucosamine pyrophosphorylase (AGX1/UAP1), cancer metabolism, multi-target approach, polypharmacology, glycosylation, UDP-hexose pyrophosphorylase, fragment-based screening

## Abstract

To minimize treatment toxicities, recent anti-cancer research efforts have switched from broad-based chemotherapy to targeted therapy, and emerging data show that altered cellular metabolism in cancerous cells can be exploited as new venues for targeted intervention. In this study, we focused on, among the altered metabolic processes in cancerous cells, altered glycosylation due to its documented roles in cancer tumorigenesis, metastasis and drug resistance. We hypothesize that the enzymes required for the biosynthesis of UDP-hexoses, glycosyl donors for glycan synthesis, could serve as therapeutic targets for cancers. Through structure-based virtual screening and kinetic assay, we identified a drug-like chemical fragment, GAL-012, that inhibit a small family of UDP-hexose pyrophosphorylases-galactose pyro-phosphorylase (GALT), UDP-glucose pyrophosphorylase (UGP2) and UDP-*N*-acetylglucosamine pyrophosphorylase (AGX1/UAP1) with an IC_50_ of 30 µM. The computational docking studies supported the interaction of GAL-012 to the binding sites of GALT at Trp190 and Ser192, UGP2 at Gly116 and Lys127, and AGX1/UAP1 at Asn327 and Lys407, respectively. One of GAL-012 derivatives GAL-012-2 also demonstrated the inhibitory activity against GALT and UGP2. Moreover, we showed that GAL-012 suppressed the growth of PC3 cells in a dose-dependent manner with an EC_50_ of 75 µM with no effects on normal skin fibroblasts at 200 µM. Western blot analysis revealed reduced expression of pAKT (Ser473), pAKT (Thr308) by 77% and 72%, respectively in the treated cells. siRNA experiments against the respective genes encoding the pyrophosphorylases were also performed and the results further validated the proposed roles in cancer growth inhibition. Finally, synergistic relationships between GAL-012 and tunicamycin, as well as bortezomib (BTZ) in killing cultured cancer cells were observed, respectively. With its unique scaffold and relatively small size, GAL-012 serves as a promising early chemotype for optimization to become a safe, effective, multi-target anti-cancer drug candidate which could be used alone or in combination with known therapeutics.

## 1. Introduction

Cancer is one of the leading causes of mortality and morbidity in both developing and developed nations. The American Cancer Society (www.cancer.org) estimates that in 2019, there will be an estimated 1,762,450 new cancer cases diagnosed and 606,880 cancer deaths in the United States alone [1]. Despite significant progress in anti-cancer therapies, some cancers continue to have poor prognosis [2,3]. Moreover, the deleterious side-effects associated with even the most advanced types of cytotoxic chemotherapies often take a serious toll on the quality of life of the patients. Recent research efforts have steadily moved from broad-based chemotherapy to targeted therapy to minimize treatment toxicities, and emerging research shows that altered cellular metabolism in cancerous cells can serve as new venues for targeted intervention.

**Figure 1:**
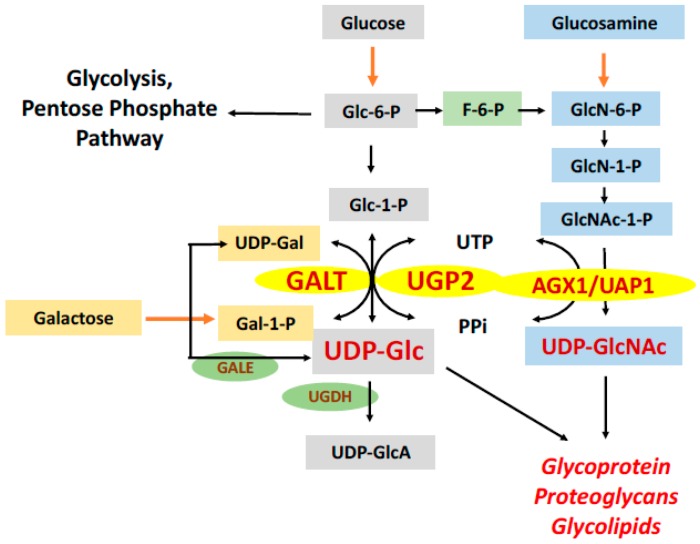
Roles of UDP-hexose pyrophosphorylases in glycan biosynthesis. Schematic representation of the roles played by the three known UDP-hexose pyrophosphorylases in the glucose (Glc) metabolic and the hexosamine biosynthetic pathways. (GALK1: galactokinase, GALT: galactose-1 phosphate-uridylyltransferase, UGP2: UDP-glucose pyrophosphorylase, UGDH: UDP-glucose 6-dehydrogenase, GALE: UDP-glucose-4-epimerase, AGX1/UAP1: UDP-*N*-acetylglucosamine pyrophosphorylase).

Metabolic reprogramming is a hallmark of cancer cells and contributes to their adaption within the tumor microenvironment and resistance to anticancer therapies [4]. Among the altered metabolic processes in cancerous cells, glycosylation has gained increasing attention because of its documented roles in cancer tumorigenesis [5,6], metastasis [7] and drug resistance [8]. For some time, abnormal cancer-specific glycoproteins have been proposed as targets for cancer control [9]. Yet, most studies focused on the enzymes [10], such as β-1,4-galactosyltransferase, that catalyze the transfers the galactosyl or glucosyl donors [11,12], with little attention was paid to the production of the substrates (i.e., UDP-hexoses), which is required for the abnormal glycoprotein and glycolipids biosynthesis in cancers. We hypothesize that targeting enzymes which produce UDP-hexoses for glycosylation could represent a novel strategy for cancer treatment. One such enzyme is galactose-1 phosphate uridylyltransferase (GALT, EC 2.7.7.12), also known as UDP-galactose pyrophosphorylase. GALT catalyzes the second step of the Leloir pathway of galactose metabolism (Figure 1). By transferring uridine monophosphate (UMP) from UDP-glucose (UDP-Glc), GALT converts galactose-1 phosphate (Gal-1P) to UDP-galactose (UDP-Gal), which is a critical substrate used to build galactose-containing glycoprotein and glycolipids. Previously, we showed that knocking down *GALT* gene expression in the HepG2 cells by siRNA led to growth inhibition of the cultured hepatoma cells [13]. We selected GALT in our studies as its complete deficiency in human causes classic galactosemia, an inborn error of metabolism [14]. It is well-documented that patients with this inherited metabolic disorder have growth retardation and aberrant glycosylation, which provides some validation of the target [15,16]. Yet, it is also known that individuals who harbor hypomorphic variations in the *GALT* genes and retain residual GALT activity are spared from the disease phenotypes [17,18]. Due to the greater demand for UDP-hexoses in cancer cells, it is therefore possible to partially inhibit GALT activity in cancers just enough to yield the desired anti-cancer effects with no detrimental effects on the normal cells.

In this report, we extend our previous study and identify a chemical fragment that inhibits GALT, GAL-012, through virtual screening. Through serendipity, we revealed that GAL-012 also inhibits two other UDP-hexose pyrophosphorylases exist in humans–UDP-glucose pyro-phosphorylase-2 (UGP2, EC 2.7.7.9) and UDP-*N*-acetylglucosamine pyrophosphorylase [AGX1/UAP1, EC 2.7.7.23] (Figure 1). We subsequently characterize this novel small molecule multi-UDP-hexose pyrophosphorylases inhibitor at structural, molecular and cell-based levels. Incidentally, AGX1/UAP1 has been proposed to be a therapeutic target for other diseases [19,20]. A GAL-012 derivative compound, GAL-012-2, was also able to inhibit GALT and UGP2, which demonstrated the potential of structural modification. The docking study revealed a different docking mode of GAL-012-2 with AGX1/UAP1. This computational prediction correlated to the insensitivity of AGX1/UAP1 to GAL-012-2 in the enzyme inhibitory assay. A preliminary structure-activity relationship (SAR) was therefore discussed. In summary, our results pave the way for the on-going optimization of the first human multi-UDP-hexose pyrophosphorylase inhibitor for therapeutic uses in human diseases.

## 2. Results

### 2.1. Fragment-Based Screening for GALT Inhibitors

**Figure 2:**
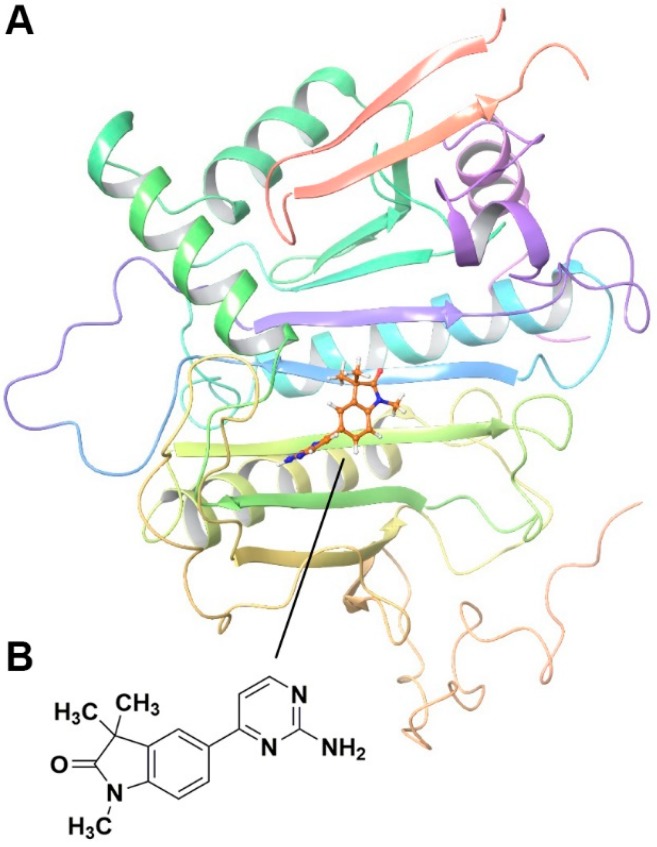

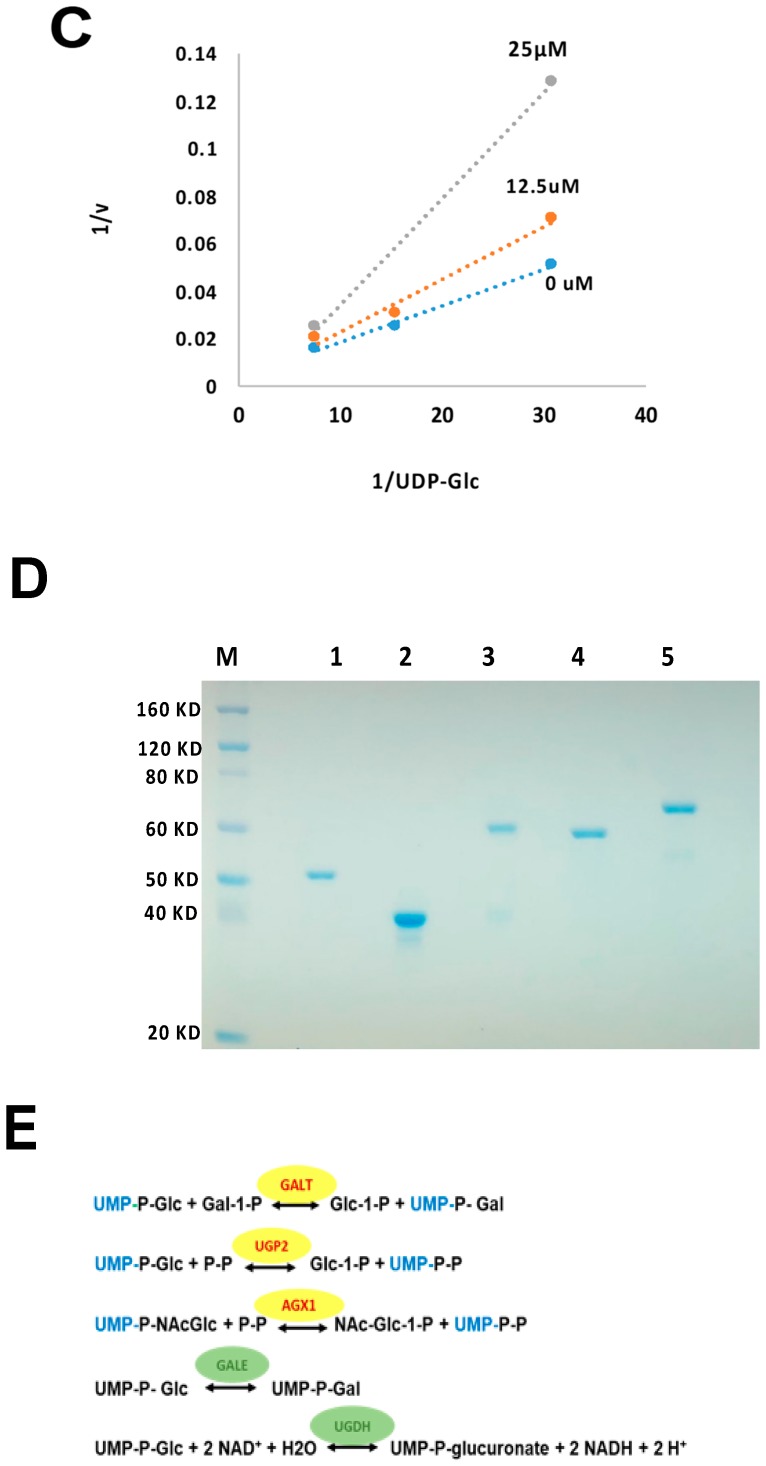
In vitro characterization of fragment GAL-012. (**A**) GAL-012 (orange) docked into GALT. (**B**) Chemical structure of fragment GAL-012. (**C**) Lineweaver-Burke plot of GALT reaction velocities as measured in varying concentrations of GAL-012. (**D**) SDS-PAGE of selected purified recombinant enzymes (M: Molecular weight marker; 1: Purified GALT; 2: Purified GALE; 3: Purified UGDH; 4: Purified UGP2; 5: Purified AGX1/UAP1.) (**E**) Reaction mechanisms of selected enzymes—GALT, UGP2, AGX1/UAP1, UGDH, GALE.

We used the recently described X-ray structure of human GALT [21] to conduct a fragment-based screening within our collection of 0.5 million diverse set of screening library (from 39 vendors) to identify potential GALT interacting fragments. Forty three fragments were identified, scored, ranked, and analyzed based on their association potentials with the active site within GALT. Figure 2A illustrated an exemplary hit compound GAL-012 docking into GALT.

### 2.2. Fragment GAL-012 Is a Competitive Inhibitor of Recombinant GALT

15 best scored fragments were dissolved in 100% DMSO to make 100 mM stocks, and 25 µM of each fragment was incubated with purified recombinant GALT for 5 min at room temperature and then added into the GALT enzymatic assay reaction mixture to initiate the reaction. GAL-012, whose structure was illustrated in Figure 2B, was the only one that significantly reduced the enzymatic activity to 63.2% when 25 µM GAL-012 was applied (Supplementary Table S4, Table 1).

Given the inhibitory action of GAL-012 on GALT, we then examined its competitive effects on the substrates of GALT: UDP-Glc and Gal-1P. Analysis of initial velocity changes at varying concentrations (12.5 µM, 25 µM and 50 µM) of GAL-012 were performed. The concentrations of each of the two GALT substrates were held constant: 0.13 mM and 0.35 mM for UDP-Glc and Gal-1P, respectively. As shown in Figure 2C, GAL-012 inhibits GALT enzymatic activity by competitive to UDP-Glc, but not Gal-1P (data not shown), with a K*_i_* around 30 µM.

### 2.3. Fragment GAL-012 Is a Novel Inhibitor of Multi-UDP-hexose Pyrophosphorylases

The above data raised the possibility that GAL-012 may target other UDP-Glc binding enzymes as well. To assess this possibility, we have prepared four other recombinant enzymes (UGP2, AGX1/UAP1, UGDH, and GALE) that recognize UDP-Glc/UDP-GlcNAc as substrates (Figure 2D) and test for their enzymatic activities in the presence and absence of three concentrations (12.5 µM, 25 µM and 50 µM) of fragment GAL-012. As shown in Table 1 and Supplementary Table S4, GAL-012 exposure resulted in reduced enzymatic activity for both UGP2 (58.46%) and AGX1/UAP1 (56.45%), and further studies on UGP2 inhibition assay revealed that GAL-012 also acted as an UDP-Glc competitive inhibitor for UGP2 (data not shown). Meanwhile, no inhibition was observed on GALE or UGDH (Supplementary Table S4). Remarkably, GALT, UGP2 and AGX1/UAP1 exhibit a pyrophosphorylase action against UDP-hexoses while the other two other enzymes (GALE and UGDH do not (Figure 2E).

### 2.4. Predicted Molecular Interactions between GAL-012 and the Respective UDP-hexose Pyrophosphorylases

**Figure 3:**
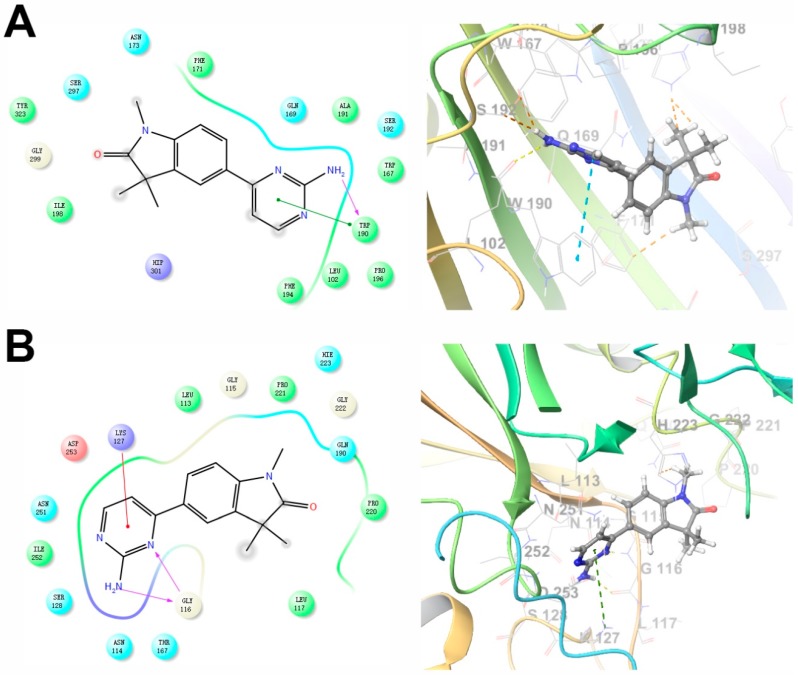

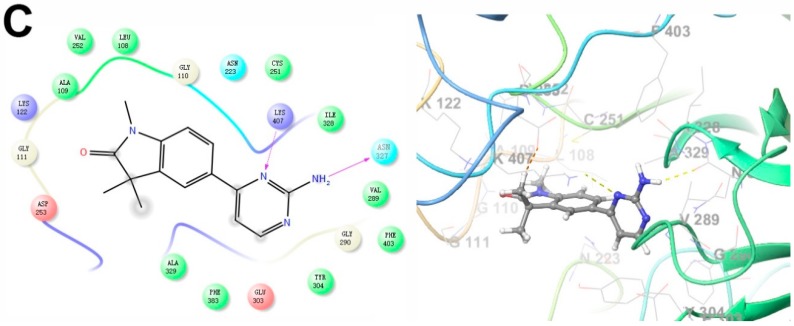
Two-dimensional (**left**) and three-dimensional (**right**) docking models of GAL-012 binding to (**A**) GALT, (**B**) UGP2 and (**C**) AGX1/UAP1. The docking experiments were performed as described previously using Glide (Schrödinger, LLC) [22]. The X-ray crystal structures of human GALT (PDB ID: 5IN3), UGP2 (PDB ID: 4R7P) and AGX1/UAP1 (PDB ID: 1JVD) was used.

To further explore binding of GAL012 to the three human UDP-hexose pyrophosphorylases (GALT, UGP2 and AGX1/UAP1), we performed docking experiments of the fragment to the respective virtual proteins structure with Glide (Schrödinger, LLC, New York, NY, USA). Potential interaction of GAL-012 within the substrate-binding domain of each enzyme was analyzed and shown in Figure 3. For GALT, we found that Trp190 and Ser192, which may be the important amino acids for substrate binding, were revealed as a predicting interaction site for hydrogen bonding with the pyrimidine amine. Gly116 and Lys127 are the two sites for the same binding of GAL-012 to UGP2. For AGX1/UAP1, Asn327 and Lys407 were considered important for hydrogen bonding, which could recognize *N*-acetyl arm of the Glc*N*Ac and Gal*N*Ac, and the output of Glide showed that both residues were binding sites for GAL-012.

### 2.5. Effects of UGP2 and AGX1/UAP1 Gene Knockdown in Cultured Cancer Cells

**Figure 4:**
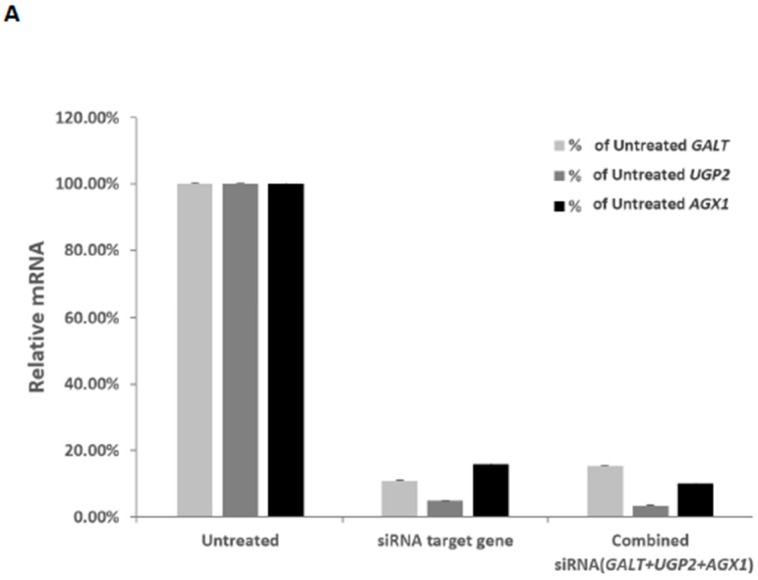

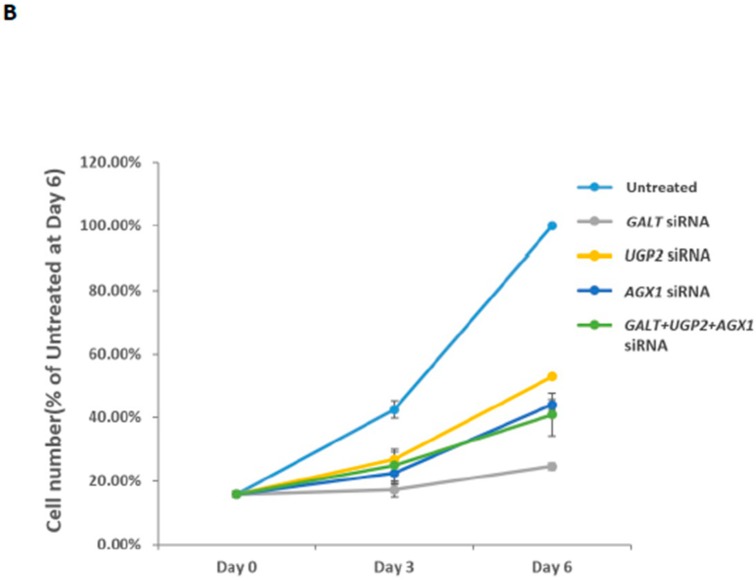
Validation of UDP-hexose pyrophorylases as anti-cancer targets by siRNA experiments. (**A**) Relative mRNA levels of *GALT*, *UGP2* and *AGX1*/*UAP1* in PC3 cells 72 h after respective siRNA transfections. Results were normalized to those found in untreated cells (100%). (**B**) Inhibition of PC3 cell growth by siRNA against *GALT*, *UGP2* and *AGX1/UAP1* genes.

Previously, we demonstrated the *GALT* gene knockdown in HepG2 cells led to growth inhibition [13]. To assess whether the two other UDP-hexose pyrophosphorylases which GAL-012 recognizes can potentially offer additional advantages in controlling cancer cell growth, we must validate the other two targets. To do so, we employed commercially-validated *UGP2* and *AGX1/UAP1* siRNAs to knockdown the respective genes in PC3 cells. In Figure 4A, we showed when we administered the respective siRNA individually, we accomplished 89%, 95% and 84% reduction of the mRNA levels of *GALT, UGP2, AGX1/UAP1*, respectively. When we added the three siRNA’s together, we saw 85%, 96% and 90% reduction, respectively. The administering of the siRNA’s also led to significant growth retardation of the PC3 cells (Figure 4B). Interestingly, *GALT* siRNA was the most effective among the three siRNA’s despite the fact that the reduction of *GALT* mRNA level was not the greatest (Figure 4A).

### 2.6. GAL-012 Derivative GAL-012-2 Inhibits GALT and UGP2

Towards a better understanding of the structural-activity relationships of GAL-012, we purchased four analogues from the commercial vendor, Otava Chemicals Ltd. (www.otavachemicals.com). As shown in Table 1, four analogues of GAL-012 were tested the inhibitory activity against GALT, UGP2 and AGX1/UAP1. One analogue, GAL-012-2 was identified to inhibit the activity of GALT and UGP2, but not AGX1/UAP1, and other analogues did not show any activity. Based on the structure difference, we can conclude that all the compounds comprise the same gem-dimethyl and *N*-methyl-substituted indolinone structure on the left side, and the difference on the right side determines the activity. GAL-012-2 used a 4-amino 3,4-unsaturated ketone to replace the pyrimidine amine of GAL-012 by maintaining a similar distance and relative position of the pyrimidyl amine to indolinone and demonstrated the similar inhibitory activity to GALT and UGP2, but not AGX1/UAP1. It seems that the pyrimidine ring is important for binding to AGX1/UAP1. However, GAL-012-1 with a thiazole ring replacing pyrimidine lost activity, suggesting the distance and relative position of the amine is important. GAL-012-3 and GAL-012-4 do not possess amine and maintain the distance of the right side of compound and they did not show any activity.

### 2.7. Docking Study of GAL-012-2 to GALT, UGP2 and AGX1/UAP1

**Figure 5:**
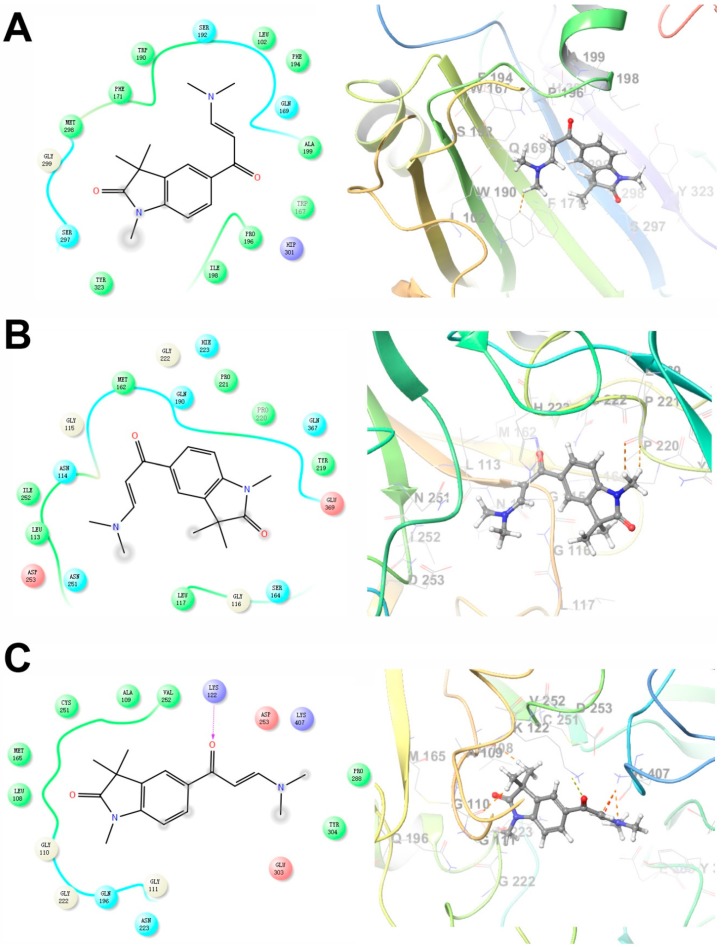

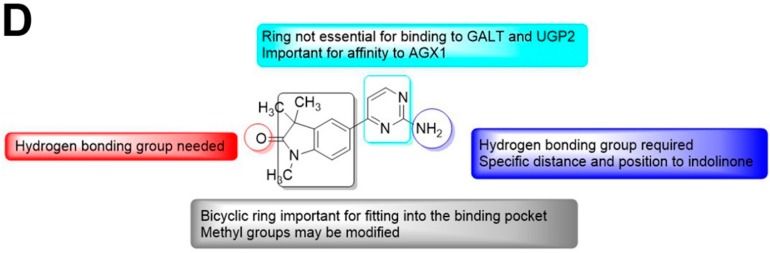
The docking models of GAL-012-2 binding to the three UDP-hexose pyrophosphorylases and Preliminary SAR analysis of GAL-012 series. Docking of GAL-012-2 to (**A**) GALT, (**B**) UGP2, and (**C**) AGX1/UAP1 were performed using Glide (Schrödinger, LLC). (**D**) SAR analysis results based on the relative activities of different analogues against the respective UDP-hexose pyrophosphorylases.

Similarly, GAL-012-2 demonstrated to adapt the same binding pockets as GAL-012 for binding to GALT and UGP2 (Figure 5A–C). However, GAL-012-2 was not able to bind to Lys407 of AGX1 without the nitrogen of pyrimidine. Therefore, the molecule has to turn over and use the dimethylamine for the interaction with Lys407 although the whole molecule can still fit into the binding pocket. The dissimilar docking result of GAL-012-2 and GAL-012 for binding to AGX1 may explain the inefficiency of GAL-012-2 in the enzyme inhibitory assay of AGX1/UAP1.

### 2.8. Preliminary SAR of GAL-012 Series of Compounds

Based on the docking models and examination results of four GAL-012 analogues, we can summarize and conclude a plausible SAR (Figure 5D): On the left side, indolinone ring seems essential for fitting into the binding pocket of proteins; the oxygen of amide is important for hydrogen bonding; the gem-dimethyl group and *N*-methyl group may be modified for better pocket fitting. On the right side, the pryrimidine amine is necessary as a hydrogen bond acceptor, and the distance and relative position from the amine to the indolinone is important; the pyrimidine ring is not essential for binding to GALT and UGP2, but affects the affinity to AGX1/UAP1.

### 2.9. Fragment GAL-012 Dose Dependently Inhibits PC3 Cell Growth

Our biochemical data above that showed fragment GAL-012 can inhibit multiple UDP-hexose pyrophosphorylases in vitro (Figure 2D and Supplementary Table S4), and we have genetically validated all three enzymes as potential targets for controlling growth of cultured cancer cells (Figure 4).

**Figure 6:**
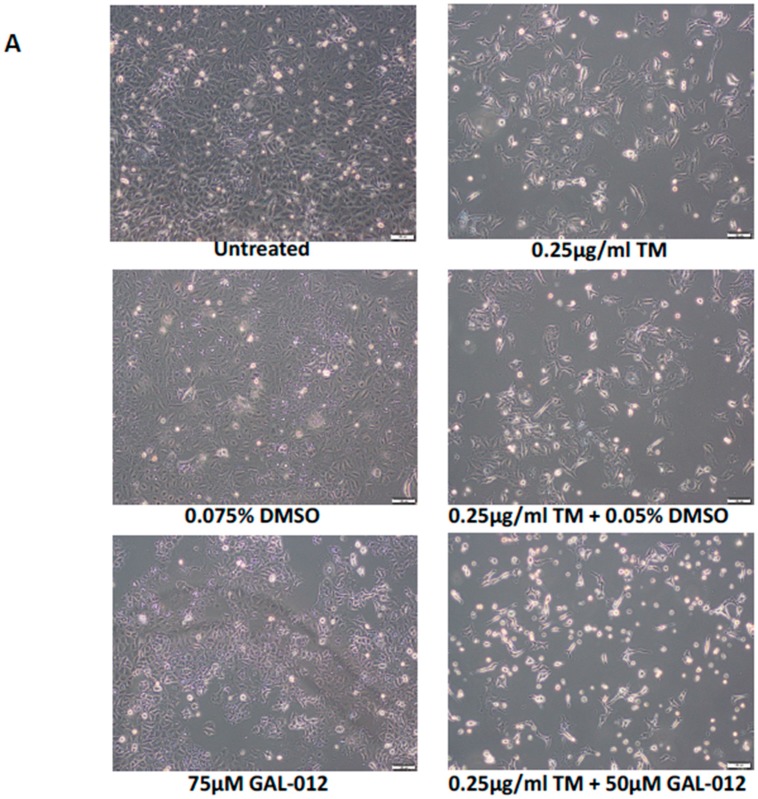

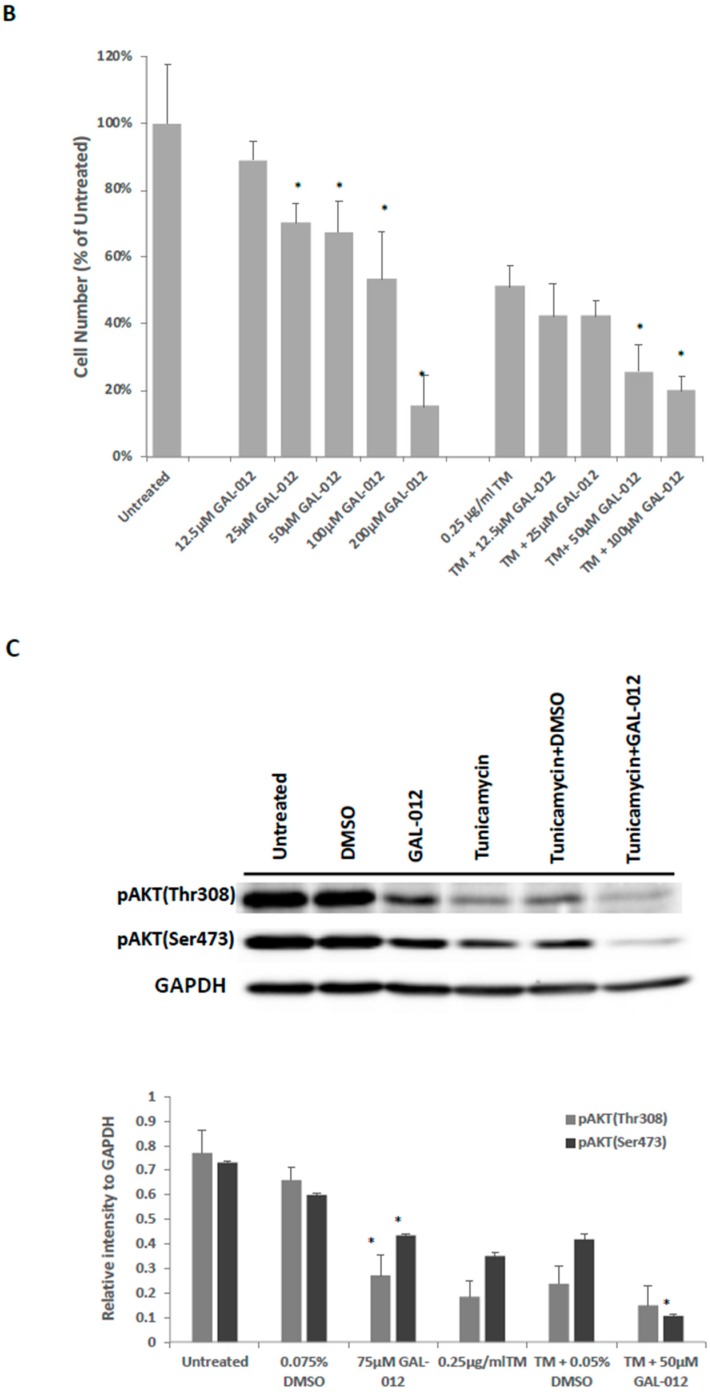

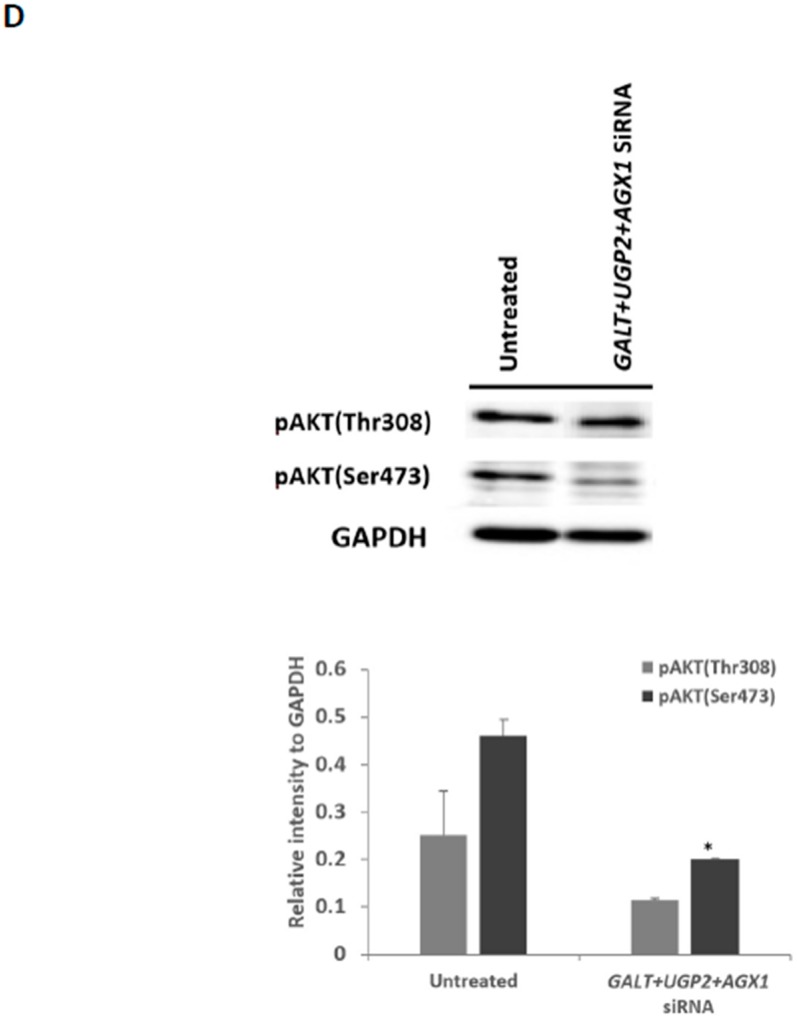
GAL-012 increased sensitivity to tunicamycin in PC3 cells: (**A**) Photomicrographs showing the growth of PC3 cells treated with varying concentrations of GAL-012 in the presence or absence of 0.25 µg/mL tunicamycin (TM) for 96 h. (**B**) Quantification of cell growth of PC3 treated with varying concentrations of GAL-012 in the presence or absence of 0.25 µg/mL tunicamycin (TM) for 96 h. (**C**) Western Blot analyses for pAKT (Thr308) and pAKT (Ser473) in PC3 cells treated with GAL-012 in the presence/absence of tunicamycin. (**D**) Western Blot analyses for pAKT (Thr308) and pAKT (Ser473) in PC3 cells treated with siRNAs against *GALT*, *UGP2* and *AGX1.UAP1*. * *p* < 0.05.

**Figure 7:**
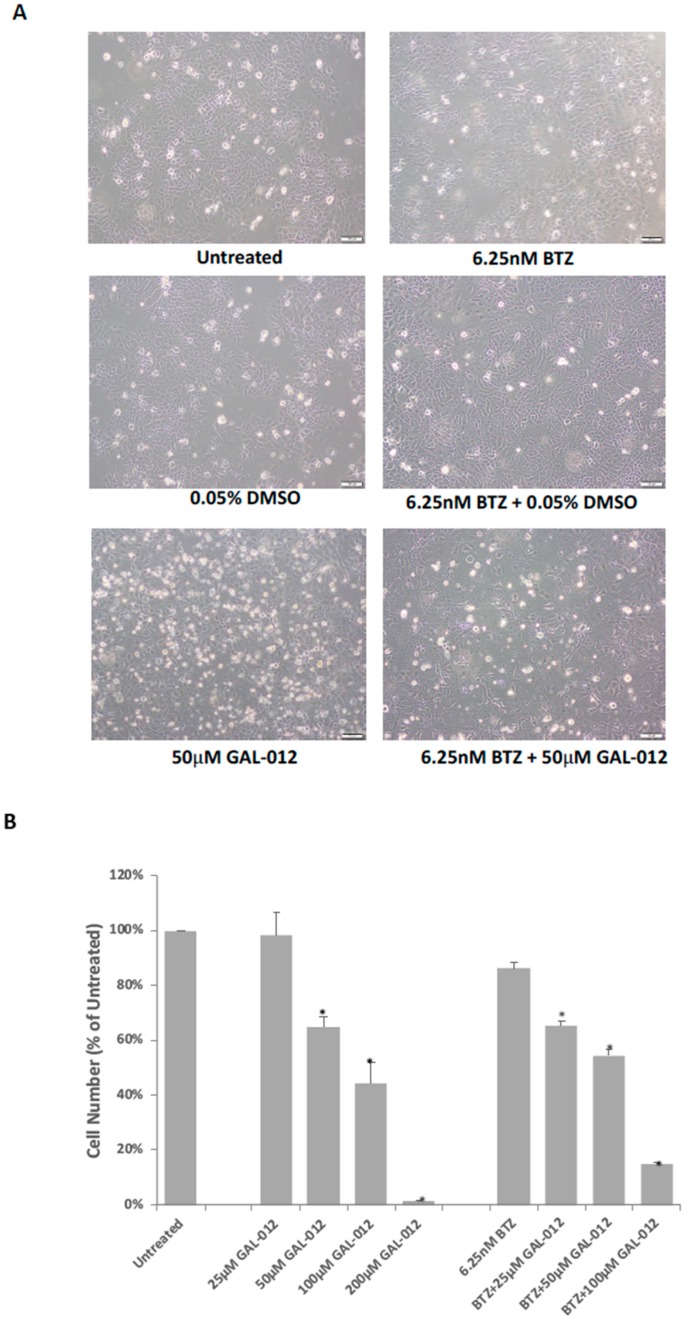
GAL-012 increased sensitivity to Bortezomib (BTZ) in HepG2 cells. (**A**) Photomicrographs showing the growth of HepG2 cells treated with varying concentrations of GAL-012 in the presence or absence of 6.25 nM BTZ for 96 h. (**B**) Quantification of cell growth of HepG2 treated with varying concentrations of GAL-012 in the presence or absence of 6.25 nM BTZ for 96 h. * *p* < 0.05.

We proceeded to further characterize GAL-012 in cell-based assays. First, we examined the effects of the fragment on the growth of cultured PC3 cells (Figure 6) and cell numbers were determined after treatment with increasing doses of GAL-012 for 4 days. In Figure 6B, we revealed that GAL-012 inhibited the proliferation of PC3 cells in a dose-dependent manner. The significant inhibitory effect of GAL-012 on PC3 cells was first appeared at 25 μM by lowering the cell count to 70.1% of untreated, while at 100 μM the cell count was lowered to 53%, suggesting an EC_50_ of 100 μM for GAL-012 on PC3 cells. Inhibition of growth by GAL-012 was also seen in HepG2 cells with an EC_50_ of 75 μM (Figure 7).

### 2.10. GAL-012 Increased Sensitivity to Tunicamycin in PC3 Cells

Itkonen and coworkers reported that *AGX1/UAP1* is over-expressed in prostate cancer cell lines, including PC3, and the over-expression is protective against inhibitors of *N*-linked glycosylation like tunicamycin (TM) [19]. In their study, the authors showed that siRNA targeting *AGX1/UAP1* gene led to increased sensitivity to tunicamycin treatment in cultured prostate cancer cells [19]. This supported our hypothesis that enzymes such as UDP-hexose pyrophosphorylases required for the biosynthesis of UDP-hexoses—the building blocks of glycans, are potential anti-cancer targets because of the higher levels of aberrant of glycosylation in cancer cells. Since GAL-012 is a multi-UDP-hexose pyrophosphorylases inhibitor, we sought if it renders PC3 cells more sensitive to tunicamycin. Figure 6B showed the synergistic relationship between GAL-012 and 0.25 µg/mL tunicamycin, a concentration that is widely used to block *N*-linked glycosylation in cultured cells [23,24].

We further explored the synergistic relationship between GAL-012 and tunicamycin at the molecular levels by examining two key players of the PI3K/AKT pro-survival/growth signaling pathway in the treated PC3 cells. We focused on the PI3K/AKT signaling pathway because it was frequently up-regulated in cancer cells, including prostate cancer cells [25,26]. Figure 6C either GAL-012 and tunicamycin treatment alone significantly reduced the level of pAKT (Thr308) and to some extent, pAKT (Ser473) (Lanes 3–5, Figure 6C). When both GAL-012 and tunicamycin were applied, we saw further decrease in pAKT (Ser473) (Lane 6, Figure 6C). The results were further supported by siRNA validation studies (Figure 6D).

### 2.11. GAL-012 Increased Sensitivity to Bortezomib (BTZ) in HepG2 Cells

The synergistic relationship between tunicamycin and GAL-012 revealed above is not only novel, but also offers support to our proposed targeting of glycosylation in cancer cells. However, tunicamycin is not an FDA-approved anti-cancer therapeutic. To determine if GAL-012 synergizes any known anti-cancer drug, we investigated if GAL-012 could enhance the sensitivity of HepG2 cells to Bortezomib (BTZ), the first therapeutic proteasome inhibitor used in human patients [27,28]. The dose of BTZ was determined by treating HepG2 cells with various concentrations of BTZ (0–50 nM) for 96 h, and 6.25 nM was selected for a drug resistant effect. As shown in Figure 7, the combination of GAL-012 and 6.25 nM BTZ led to a greater inhibition on HepG2 cell growth than with either agent alone. Treatment of cells with 6.25 nM BTZ for 4 days did not significantly reduce the cell survival (*p* > 0.05 vs. control) while the addition of 25 μM GAL-012 caused a 35% reduction in the cell number (*p* < 0.05 vs. control).

## 3. Discussion

Despite on-going advances in cancer research, cancer-related mortality and morbidity continue to pose serious threats to human health and well-being. For instance, hepatocellular carcinoma (HCC) is one of the most prevalent cancers and the third leading cause of cancer-related deaths worldwide [29,30,31]. Although many therapeutic strategies, such as surgical resection, liver transplantation, and radiofrequency ablation, have been employed, its prognosis remains unfavorable [32,33]. With regards to cancers that have relatively better prognosis, current treatments often render significant side-effects. For example, treatment of prostate cancers—one of the most common and more “curable” cancers if diagnosed early enough, frequently result in sexual, bowel, and urinary dysfunctions [34,35,36]. Hence, better treatment options are urgently needed for all cancers.

Metabolic reprogramming contributes to tumor development, metastasis and drug resistance. Therefore, others and we have proposed to exploit the metabolic liabilities to treat cancers [13,37,38,39,40,41,42]. In prior studies where we focused on aberrant glycosylation in cancer cells, we demonstrated that disruption of the galactose metabolism pathway by siRNAs against GALT inhibited the growth of HepG2 cells in culture and proposed that GALT is a novel therapeutic target for HCC [13]. Those preliminary studies suggested small molecular inhibitors target GALT could be a start for effective anti-cancer drugs development. The discovery of hits by high-throughput screening (HTS) is costly and time-consuming and demands large for labor. On the other hand, virtual screening requires less time and investment has been widely used for facilitating drug discovery [43]. Besides, structure-based drug design (SBDD) techniques are known to be powerful approaches to identify and optimize hit compounds as drug candidates [44]. Here we carried out a structure-based virtual screening campaign and kinetic assays to identify a novel chemical fragment, GAL-012 (Figure 2), that not only inhibits GALT, but also targets other two UDP-hexose pyrophosphorylases, UGP2 and AGX1/UAP1 (Supplementary Table S4, Figure 3 and Table 1). To the best of our knowledge, GALT, UGP2 and AGX1/UAP1 are the only known *human* UDP-hexose pyrophosphorylases. While GAL-012 functions as a chemical probe to understand that structural biology of human UDP-hexose pyro-phosphorylases, our results suggested that it can be treated as an early generation, therapeutic multi-UDP-hexose pyrophosphorylases inhibitor. In order to argue that ability of GAL-012 to inhibit multi-enzymes offer additional advantages towards the targeting of a single enzyme, we must show that the enzymes involved are valid therapeutic targets, as well as the fact that multi-target approach is sound.

Indeed, both *UGP2* and *AGX1/UAP1* have been shown to play a role in cancer growth. A recent proteomics study showed that the *UGP2* expression levels were significantly higher in some malignant tumors or malignant cells than in normal tissue or cells [45]. UGP2 overexpression has also been demonstrated to promote cell migration and invasion, as well as enhanced glycogenesis in vitro, which provides UGP2 as a valuable target in cancer treatment [46,47]. As for AGX1/UAP1, it has been proposed to be a therapeutic target for other diseases such as diabetes and prostate cancers [19,20]. To be rigorous, we genetically validated both targets in prostate cancer cell line PC3 in this study (Figure 4).

As for multi-target approaches (also known as polypharmacology), it is gaining attention in the field of drug discovery, especially for complex diseases like cancers because of significant advantages [48,49,50,51,52,53,54]. To begin with, if we can address multiple targets with just one drug, we will have less issues with pharmacokinetics and/or drug resistance. In our case, our proposed targeting GALT, UGP2, and AGX1/UAP1, which are key enzymes in involved with the production of glycoproteins/glycolipids is also unique because it is the first time that targets implicated in glycan biosynthesis have been chosen for multi-targeting approach for any disease. Moreover, since all three targets operate on similar metabolic pathways that are closely linked to each other (Figure 1), GAL-012 can potentially offer additional synergistic effects [55]. Although we did not perform any extensive off-target analysis in this study, we believe GAL-012 offers sufficiently good selectivity for the three targets since it shows no inhibition against the other two enzymes that use UDP-glucose as substrate–GALE and UGDH (Supplementary Table S4).

Still, we investigated whether co-treatment with GAL-012 with other known drugs—an experimental drug (tunicamycin) and an FDA-approved anticancer drug [Bortezomib (BTZ)], will lead to increased killing of the cultured cancer cells. As shown in Figure 6 and Figure 7, we saw synergistic effects of GAL-012 with tunicamycin and BTZ for PC3 and HepG2 cells, respectively. Even though these results are preliminary in nature, it suggested that GAL-012 (or its more potent, drug-like derivatives) could be used in combination with other anti-cancer drugs.

In this study, we did not measure changes in UDP-hexoses or glycosylation in cancer cells treated by GAL-012. This is because such changes are well-documented in clinical studies of patients with GALT-deficiency classic galactosemia. Indeed, classic galactosemia is known as a secondary congenital disorder of glycosylation (CDG) because of glycosylation defects detected in patient cells [15,56,57,58,59,60,61,62], which were attributed to decreased UDP-hexose contents [63,64,65,66]. Similarly, Itkonen and coworkers showed that siRNA targeting *AGX1/UAP1* gene in PC3 cells led to increased sensitivity to tunicamycin treatment in cultured prostate cancer cells, as well as over 60% decrease in UGP-GlcNAc and UDP-GalNAc [19]. Therefore, there is already ample of evidence in literature that targeting UDP-hexose pyrophosphorylases will alter cellular UDP-hexose contents and glycosylation, which are critical for cancer cell survival.

Although the potency of GAL-012 remains to be optimized, one must evaluate the current data in the context of the fact that it is an unmodified hit fragment from our virtual screening, and one of its derivatives also showed activity against GALT and UGP2, demonstrating the potential of structural modification. The structure of hit compound GAL-012 is also an appropriate starting point for lead optimization. The indolinone moiety in GAL-012 is very common in bioactive compounds [67], and pyrimidine is the basic component in nucleosides. Because of its relatively small size, there are ample of opportunities to optimize this fragment based on high-precision docking experiments similar to the ones we performed in Figure 3 or X-ray co-crystallography studies in the future.

## 4. Methods

### 4.1. In Silico Fragment-Based Screening for GALT Inhibitors

The X-ray crystal structure of GALT (PDB ID: 5IN3) [21] was retrieved from Protein Data Bank (https://www.rcsb.org). A 0.5 million diverse set of fragments from 39 vendors along with 3000 University of Utah Center for Investigational Therapeutics (CIT) in-house collection were chosen for computational screening using ICM Suite (Molsoft, San Diego, CA, USA). The fragment library enumeration associated rule of 3 (RO3) [68] criteria with a molecular weight cut off of >150 to <300 was submitted for virtual ligand functionality screening against the crystal structure of GALT target. 15 fragment hits from both libraries were selected based on consensus scores and binding energies, which led to the identification of promising low micro molar fragments as GALT inhibitor scaffolds that are within the RO3 chemical space suitable for further optimization.

### 4.2. Expression Cloning of Human UDP-hexose Binding Enzymes: GALT, UDP-galactose 4′ Epimerase (GALE), UDP-Glc Dehydrogenase (UGDH), UGP2, and AGX1/UAP1

The plasmids used for overexpression of human *GALT* and *UGP2* were previously prepared in our laboratory. [69,70] The cDNA sequences of human *AGX1/UAP1, GALE* and *UGDH* and were retrieved from NCBI Genbank. The reference sequences are NM_001324116.1 for AGX1/UAP1, NM_000403.3 for *GALE* and NM_003359.4 for *UGDH*, respectively. In order to clone the full-length gene encoding *AGX1*/*UAP1*, *GALE* and *UGDH*, RT-polymerase chain reaction (PCR) was performed on RNA extracted from HepG2 cells using specific oligonucleotides (Supplementary Table S1). A single full-length PCR product for each gene was obtained. The PCR fragment was purified from the agarose gel and double digested with *BamH*I and *Xho*I restriction enzymes. The digested product was ligated with pET30A(+) cloning vector at 16 °C overnight. The ligated product was transformed into DH5α competent cells. The positive clones were confirmed by colony PCR and Sanger sequencing. A single open reading frame for each gene was isolated. No variation in the coding sequence was found from the sequence described on NCBI Genbank.

### 4.3. Over-Expression, Affinity Purification, and Quantification of Recombinant GALT, GALE, UGDH, UGP2, and AGX1/UAP1 Enzymes

All expression plasmids containing the designed insert was transformed respectively into *Escherichia coli* (*E. coli*) HMS174 (DE3) cells (Novagen, Gibbstown, NJ, USA). Isopropyl β-D-1-thiogalactopyranoside (IPTG) was added at a final concentration of 0.1 mM to the bacterial cell culture in LB kanamycin upon reaching OD_600_ = 0.6 at 37 °C to induce overexpression for 22 h at 22 °C.

Purification for each recombinant protein was conducted at 22 °C. Briefly, cell pellets were resuspended in lysis buffer (50 mM NaH_2_PO4, 300 mM NaCl, 10 mM imidazole, pH 8). Cells were then lysed using a cell disruptor (model, 749540-0000, Kimble Chase, Rockwood, TN, USA) for 10 min at 4 °C and clarified by centrifugation, and the lysate was loaded onto a chromatography column containing Nickel affinity resin (Ni-NTA) (Qiagen, Valencia, CA, USA). The resin was washed, and bound recombinant protein was eluted using an imidazole concentration gradient. Final purified enzyme was concentrated to 1 mg/mL, dialyzed into phosphate-buffered saline (PBS, company, Genesee Scientific, San Diego, CA, USA), aliquoted, and stored frozen at −80 °C.

### 4.4. Enzyme Activity Assays for Recombinant Human GALT, GALE, UGDH, UGP2 and AGX1/UAP1 Enzymes

#### 4.4.1. GALT, UGP2, AGX1/UAP1 Enzymatic Activities Measurements

Procedures of enzymatic assays for purified recombinant GALT, UGP2 and AGX1/UAP1 were described in our previous publications [63,70].

#### 4.4.2. GALE Enzyme Activity Measurements

Human GALE enzyme activity was calculated from the conversion of UDP-Gal to UDP-Glc. Briefly, a reaction mixture consisting of 100 mM glycine buffer (pH 8.8), 0.2 mM UDP-Gal, 30 mM NAD^+^ and 0.025 U UDP-glucose dehydrogenase was freshly prepared before the assay. The formation of NADH was quantified by monitoring change in absorbance of the reaction mixture at 340 nm for 15 min using a BioTek plate reader (BioTek, Winooski, VT, USA). The relationship between increase in NADH production and UDP-Glc released was quantified using the Beer-Lambert equation.

#### 4.4.3. UGDH Enzymatic Activity Measurements

UGDH activity was measured in a mixture of 100 mM glycine buffer (pH 8.7), 0.2 mM UDP-Glc and 1 mM β-NAD at 22 °C. One unit of enzyme was defined as the amount of enzyme required to reduce 2 μmol of NAD^+^/min at pH 8.7 at 22 °C.

### 4.5. Inhibition Kinetics of Fragment GAL-012 and Derivatives

To determine if GAL-012 or its derivatives inhibits recombinant GALT and other selected UDP-hexose binding enzymes, GAL-012 was used as an example. The fragment was incubated, at various concentrations, for 5 min with the respective enzyme before the initiation of the corresponding enzymatic reaction. Same concentration of DMSO used as controls.

To determine if fragment GAL-012 is competitive for UDP-glucose in the GALT reaction, varying concentrations of UDP-glucose are present in the GALT reactions in the presence of a fixed quantity of GAL-012. The initial velocity of the reaction was measured by monitoring the change of absorbance at 340 nm, and was plotted against the substrate concentration. Curve-fitting was accomplished using the equation V = V_max_ S/(K*_M_* + S) by Sigma Plot 10.0 (Systat Software Inc., San Jose, CA, USA, 2019), and the V_max_ and K*_M_* were calculated from the above-stated equation. K*_i_* values were calculated from equations, as described elsewhere [63].

### 4.6. Molecular Docking Experiments of GAL-012 and GAL-012-2 to GALT, UGP2 and AGX1/UAP1

In order to study the binding mode of GAL-012 and GAL-012-2 to the human GALT, UGP2 and AGX1/UAP1, ligand-protein docking studies were performed as previously using Glide (Schrödinger, LLC) [22]. The X-ray crystal structures of human GALT (PDB ID: 5IN3), UGP2 (PDB ID: 4R7P) [71] and AGX1/UAP1 (PDB ID: 1JVD) [72] were retrieved from PDB, and each protein was prepared by Schrödinger 2015. Grids generation was set up on proteins and saved as job ZIP format. 2D structure of GAL-012 was built using ChemDraw program (ChemDraw Ultra 10.0, CambridgeSoft, Cambridge, MA, USA, 2019), and then converted to SDF file format. The ligand was prepared with Schrödinger 2015, without tautomers and stereoisomers. For molecular docking, precision was defined as SP standard. All figures with structure representations were produced using PyMol and Maestro (Schrödinger, LLC).

### 4.7. Cell-Based Studies of siRNA-Treated Culture Cells

#### 4.7.1. siRNA Transfection

The siRNAs for *GALT, UGP2* and *AGX1*, respectively were synthesized from Invitrogen and were transfected into cultured cells using Lipofectamine RNAiMAX Reagent (Invitrogen, Carlsbad, CA, USA) at 10 nM according to the manufacturer’s protocol on the same day. Nucleotide sequence of the siRNAs are shown in Supplementary Table S2.

#### 4.7.2. RNA Isolation and Reverse Transcription Polymerase Chain Reaction (RT-PCR)

PC3 cells were seeded and transfected with the respective siRNAs at a density of 2 × 10^4^ cells/well onto a 24-well culture plate. After 72 h, cells were collected and total RNA was extracted by using Quick-RNA Microprep Kit (ZYMO, Irvine, CA, USA). For assay of gene expression, RNA was reverse-transcribed by using the high capacity cDNA reverse transcription kits (Applied Biosystems, Foster City, CA, USA). Quantitative PCR was performed with PowerUp™ SYBR™ Green Master Mix (Applied Biosystems) on ABI 7500 Fast Real-Time PCR system. The primers used were listed in Supplementary Table S3. Relative mRNA levels were normalized to GAPDH and calculated with the comparative CT Method (ΔΔCT Method). All transfections were performed in triplicates and for each biological replicate at least four technical replicates of the qPCR assay were performed.

#### 4.7.3. Cell Viability Assay

Cell viability was evaluated by using MTT (3-(4,5-dimethylthiazol-2-yl)-2,5-diphenyl-tetrazolium bromide) assay. In brief, cells were seeded and transfected with siRNA at a density of 4 × 10^3^ cells/well in 24-well plates and incubated for 0, 3 days and 6 days. For 6 days’ incubation, a second transfection was carried out at 72 h. At each time point, MTT (50 µL, final concentration is 0.5 µg/mL) was added into each well and incubated for 4 h at 37 °C. Then 500 µL SDS-HCl solution (10% SDS in 0.1 M HCl) was added and incubated for another 4 h at 37 °C. Sample was mixed and absorbance was obtained at 570 nm. All transfections were performed in triplicates. Cells without transfection at day 6 were used as control with 100% viability.

#### 4.7.4. PI3K/AKT Signaling Pathway in siRNA-Treated Cultured Cells

Assessment of PI3K/AKT signaling in siRNA-treated cultured cells were evaluated by Western Blot analysis. PC3 cells were seeded and transfected with siRNA at a density of 8 × 10^4^ cells/well in 6-well plates and a second transfection was carried out at 72 h later. Cells were collected at day 4 in RIPA buffer containing 50 mM Tris-HCl, 150 mM NaCl, 0.5% deoxycholate (DOC), 1% NP-40, 0.1% SDS, with protease and phosphatase inhibitors. After centrifugation at 13,000× *g* for 10 min, samples containing 40 µg of protein were resolved by SDS-PAGE and proteins were transferred to nitrocellulose membrane (Bio-Rad Laboratories, Hercules, CA, USA), blocked in 5% non-fat dried milk, and incubated with the primary antibodies for pAKT (Thr308) (Cell Signaling), pAKT (Ser473) (Cell Signaling), and GAPDH (Cell Signaling, Danvers, MA, USA), respectively overnight at 4 °C. The blots were washed with wash buffer and secondary antibody (Cell Signaling) were applied for 1 h at 22 °C. Then the blots were developed with an ECL system (GeneTex, Irvine, CA, USA) and scanned with iBright image system (Thermo Fisher Scientific, Waltham, MA, USA). Analysis was performed on iBright Analysis Software (Thermo Fisher Scientific, Waltham, MA, USA).

### 4.8. Cell-Based Characterization of GAL-012-Treated Cultured Cells

PC3 cells were maintained in 24-well plates in F-12K medium supplemented with 10% fetal bovine serum (FBS), 100 U/mL penicillin, and 100 µg/mL streptomycin (Gibco BRL, Thermo Fisher Scientific, Waltham, MA, USA).

#### 4.8.1. Gross Examination of Growth Inhibition

GAL-012 were incubated with PC3 at concentration of 12.5 µM, 25 μM, 50 μM, 100 μM and 200 μM. Same concentration of solvent DMSO were incubated as vehicle controls. After 4 days treatment, the cells were then trypsinized from the respective wells and counted with a hemocytometer for growth assessment. All experiments at each concentration of GAL-012 had three replicates and were repeated three times.

#### 4.8.2. PI3K/AKT Signaling Pathway in GAL-012-Treated Cultured Cells

PI3K/AKT Signaling upon GAL-012 treatment in PC3 cells were evaluated by Western Blot analysis. PC3 cells were treated in the presence and absence of 75 μM GAL-012 and the same concentration of solvent DMSO were incubated as vehicle controls. After 4 days treatment, the cells were collected and the procedure of Western blot were conducted as described as above. Analysis was performed on iBright Analysis Software (Thermo Fisher Scientific).

### 4.9. Combined GAL-012 and Tunicamycin Treatment of PC3 Cells

Tunicamycin (TM, Cayman Chemicals, Ann Arbor, MI, USA) was dissolved in deionized water and stored at −20 °C, and then thawed and diluted in media for cell culture experiments before use. PC3 cells were maintained in 24 well plates in F-12K medium supplemented with 10% FBS, 100 U/mL penicillin, and 100 µg/mL streptomycin (Gibco BRL). GAL-012 was incubated with PC3 at concentration of 12.5 μM, 25 μM, 50 μM and 100 μM with 0.25 μg/mL TM. After 4 days treatment, the cells were then trypsinized from the respective wells and counted with a hemocytometer for toxicity assessment. All experiments at each concentration of GAL-012 had three replicates and were repeated three times.

### 4.10. Combined GAL-012 and Bortezomib (BTZ) Treatment HepG2 Cells

Bortezomib (Millennium, Cambridge, MA, USA) was dissolved in deionized water and stored at −20 °C, and then thawed and diluted in media for cell-culture experiments before use. HepG2 cells were maintained in 24-well plates in DMEM medium supplemented with 10% FBS, 100 U/mL penicillin, and 100μg/mL streptomycin (Gibco BRL). GAL-012 was incubated with HepG2 at concentrations of 12.5 μM, 25 μM, 50 μM and 100 μM, respectively in the presence of 6.25 nM BTZ. Same concentration of solvent DMSO were incubated as vehicle control. Four days after, the cells were then trypsinized from the respective wells and counted with a hemocytometer for toxicity assessment. All experiments at each concentration of GAL-012 had three replicates and were repeated three times.

### 4.11. Statistical Analysis

Data were presented as mean ± SEM and analyzed using the two-sided unpaired Student’s *t* test. A *p* value of <0.05 was considered as statistically significant.

## Figures and Tables

**Table 1:** Inhibitory Activity of GAL-012 series to GALT, UGP2 and AGX1/UAP1.

Compound	Structure	Enzyme Inhibitory Activity		
		GALT	UGP2	AGX1/UAP1
GAL-012	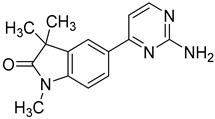	63.2 ± 2.6%	58.5 ± 6.5%	56.4 ± 5.3%
GAL-012-1	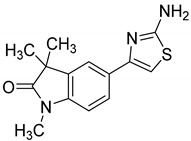	113.4 ± 0.8%	111.9 ± 2.1%	119 ± 1.8%
GAL-012-2	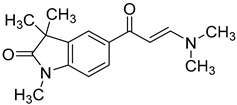	63.4 ± 2.3%	63.8 ± 4%	107.9 ± 4.7%
GAL-012-3	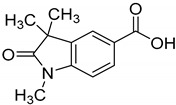	121.2 ± 14.3%	106.1 ± 4%	104 ± 8.9%
GAL-012-4	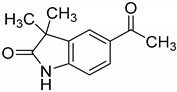	125.8 ± 7.9%	103.2 ± 4.5%	107.7 ± 5.3%

## Supplementary Materials

The following are available online. Table S1: Nucleotide sequence of primers used for GALE, UGDH and AGX1/UAP1 Cloning. Table S2: siRNA nucleotide sequence. Table S3: Nucleotide sequence of primers used for RT-PCR. Table S4: Enzymatic Activity (25 μM GAL-012) as % of vehicle (DMSO).

## References

[1] Siegel R.L., Miller K.D., Jemal A. (2018). Cancer statistics, 2018. CA Cancer J. Clin..

[2] Islami F., Miller K.D., Siegel R.L., Fedewa S.A., Ward E.M., Jemal A. (2017). Disparities in liver cancer occurrence in the United States by race/ethnicity and state. CA Cancer J. Clin..

[3] Moris D., Chakedis J., Sun S.H., Spolverato G., Tsilimigras D.I., Ntanasis-Stathopoulos I., Spartalis E., Pawlik T.M. (2018). Management, outcomes, and prognostic factors of ruptured hepatocellular carcinoma: A systematic review. J. Surg. Oncol..

[4] Pavlova N.N., Thompson C.B. (2016). The Emerging Hallmarks of Cancer Metabolism. Cell Metab..

[5] Wang M., Zhu J., Lubman D.M., Gao C. (2019). Aberrant glycosylation and cancer biomarker discovery: A promising and thorny journey. Clin. Chem. Lab. Med..

[6] Pinho S.S., Reis C.A. (2015). Glycosylation in cancer: Mechanisms and clinical implications. Nat. Rev. Cancer.

[7] Josic D., Martinovic T., Pavelic K. (2019). Glycosylation and metastases. Electrophoresis.

[8] Very N., Lefebvre T., El Yazidi-Belkoura I. (2018). Drug resistance related to aberrant glycosylation in colorectal cancer. Oncotarget.

[9] Fuster M.M., Esko J.D. (2005). The sweet and sour of cancer: Glycans as novel therapeutic targets. Nat. Rev. Cancer.

[10] Lynch T.P., Reginato M.J. (2011). O-GlcNAc transferase: A sweet new cancer target. Cell Cycle.

[11] Chatterjee S.B., Hou J., Ratnam Bandaru V.V., Pezhouh M.K., Syed Rifat Mannan A.A., Sharma R. (2019). Lactosylceramide synthase beta-1,4-GalT-V: A novel target for the diagnosis and therapy of human colorectal cancer. Biochem. Biophys. Res. Commun..

[12] Brockhausen I., Benn M., Bhat S., Marone S., Riley J.G., Montoya-Peleaz P., Vlahakis J.Z., Paulsen H., Schutzbach J.S., Szarek W.A. (2006). UDP-Gal: GlcNAc-R beta1,4-galactosyltransferase—A target enzyme for drug design. Acceptor specificity and inhibition of the enzyme. Glycoconj. J..

[13] Tang M., Etokidem E., Lai K. (2016). The Leloir Pathway of Galactose Metabolism-A Novel Therapeutic Target for Hepatocellular Carcinoma. Anticancer Res..

[14] Isselbacher K.J., Anderson E.P., Kurahashi K., Kalckar H.M. (1956). Congenital galactosemia, a single enzymatic block in galactose metabolism. Science.

[15] Maratha A., Stockmann H., Coss K.P., Gozalbo M.E.R., Knerr I., Fitzgibbon M., McVeigh T.P., Foley P., Moss C., Colhoun H.O. (2016). Classical galactosemia: Novel insights in IgG N-glycosylation and N-glycan biosynthesis. Eur. J. Hum. Genet..

[16] Waisbren S.E., Potter N.L., Gordon C.M., Green R.C., Greenstein P., Gubbels C.S., Rubio-Gozalbo E., Schomer D., Welt C., Anastasoaie V. (2012). The adult galactosemic phenotype. J. Inherit. Metab. Dis..

[17] Lai K., Langley S.D., Singh R.H., Dembure P.P., Hjelm L.N., Elsas L.J. (1996). A prevalent mutation for galactosemia among black Americans. J. Pediatr..

[18] Yuzyuk T., Balakrishnan B., Schwarz E.L., De Biase I., Hobert J., Longo N., Mao R., Lai K., Pasquali M. (2018). Effect of genotype on galactose-1-phosphate in classic galactosemia patients. Mol. Genet. Metab..

[19] Itkonen H.M., Engedal N., Babaie E., Luhr M., Guldvik I.J., Minner S., Hohloch J., Tsourlakis M.C., Schlomm T., Mills I.G. (2015). UAP1 is overexpressed in prostate cancer and is protective against inhibitors of N-linked glycosylation. Oncogene.

[20] Yarema K.I., Saeui C.T., Quinones-Hinojosa A., Shan S.R. (2018). Use of UAP Inhibitors to Inhibit Flux through the Hexosamine Biosynthetic Pathway. U.S. Patent.

[21] McCorvie T.J., Kopec J., Pey A.L., Fitzpatrick F., Patel D., Chalk R., Shrestha L., Yue W.W. (2016). Molecular basis of classic galactosemia from the structure of human galactose 1-phosphate uridylyltransferase. Hum. Mol. Genet..

[22] Ye J., Yang X., Xu M., Chan P.K., Ma C. (2019). Novel N-Substituted oseltamivir derivatives as potent influenza neuraminidase inhibitors: Design, synthesis, biological evaluation, ADME prediction and molecular docking studies. Eur. J. Med. Chem..

[23] Surani M.A. (1979). Glycoprotein synthesis and inhibition of glycosylation by tunicamycin in preimplantation mouse embryos: Compaction and trophoblast adhesion. Cell.

[24] Leavitt R., Schlesinger S., Kornfeld S. (1977). Tunicamycin inhibits glycosylation and multiplication of Sindbis and vesicular stomatitis viruses. J. Virol..

[25] Toren P., Zoubeidi A. (2014). Targeting the PI3K/Akt pathway in prostate cancer: Challenges and opportunities (review). Int. J. Oncol..

[26] Yuan T.L., Cantley L.C. (2008). PI3K pathway alterations in cancer: Variations on a theme. Oncogene.

[27] Chen D., Frezza M., Schmitt S., Kanwar J., Dou Q.P. (2011). Bortezomib as the first proteasome inhibitor anticancer drug: Current status and future perspectives. Curr. Cancer Drug Targets.

[28] Kim G.P., Mahoney M.R., Szydlo D., Mok T.S., Marshke R., Holen K., Picus J., Boyer M., Pitot H.C., Rubin J. (2012). An international, multicenter phase II trial of bortezomib in patients with hepatocellular carcinoma. Investig. New Drugs.

[29] Siegel R.L., Miller K.D., Jemal A. (2017). Cancer Statistics, 2017. CA Cancer J. Clin..

[30] Islami F., Goding Sauer A., Miller K.D., Siegel R.L., Fedewa S.A., Jacobs E.J., McCullough M.L., Patel A.V., Ma J., Soerjomataram I. (2018). Proportion and number of cancer cases and deaths attributable to potentially modifiable risk factors in the United States. CA Cancer J. Clin..

[31] Torre L.A., Bray F., Siegel R.L., Ferlay J., Lortet-Tieulent J., Jemal A. (2015). Global cancer statistics, 2012. CA Cancer J. Clin..

[32] Lafaro K.J., Demirjian A.N., Pawlik T.M. (2015). Epidemiology of hepatocellular carcinoma. Surg. Oncol. Clin. N. Am..

[33] Miller K.D., Siegel R.L., Lin C.C., Mariotto A.B., Kramer J.L., Rowland J.H., Stein K.D., Alteri R., Jemal A. (2016). Cancer treatment and survivorship statistics, 2016. CA Cancer J. Clin..

[34] Peyronnet B., Brucker B.M. (2018). Management of Overactive Bladder Symptoms after Radical Prostatectomy. Curr. Urol. Rep..

[35] Garcia-Baquero R., Fernandez-Avila C.M., Alvarez-Ossorio J.L. (2018). Functional results in the treatment of localized prostate cancer. An updated literature review. Rev. Int. Androl..

[36] Gaither T.W., Awad M.A., Osterberg E.C., Murphy G.P., Allen I.E., Chang A., Rosen R.C., Breyer B.N. (2017). The Natural History of Erectile Dysfunction after Prostatic Radiotherapy: A Systematic Review and Meta-Analysis. J. Sex. Med..

[37] Vander Heiden M.G. (2011). Targeting cancer metabolism: A therapeutic window opens. Nat. Rev. Drug Discov..

[38] Zhang W., Zhang S.L., Hu X., Tam K.Y. (2015). Targeting Tumor Metabolism for Cancer Treatment: Is Pyruvate Dehydrogenase Kinases (PDKs) a Viable Anticancer Target?. Int. J. Biol. Sci..

[39] Wang G., Wang J.J., Yin P.H., Xu K., Wang Y.Z., Shi F., Gao J., Fu X.L. (2019). Strategies to target energy metabolism in consensus molecular subtype 3 along with Kirsten rat sarcoma viral oncogene homolog mutations for colorectal cancer therapy. J. Cell Physiol..

[40] Pope E.D., Kimbrough E.O., Vemireddy L.P., Surapaneni P.K., Copland J.A., Mody K. (2019). Aberrant lipid metabolism as a therapeutic target in liver cancer. Expert Opin. Ther. Targets.

[41] Ocana M.C., Martinez-Poveda B., Quesada A.R., Medina M.A. (2019). Metabolism within the tumor microenvironment and its implication on cancer progression: An ongoing therapeutic target. Med. Res. Rev..

[42] Lee M., Ko H., Yun M. (2018). Cancer Metabolism as a Mechanism of Treatment Resistance and Potential Therapeutic Target in Hepatocellular Carcinoma. Yonsei. Med. J..

[43] Jorgensen W.L. (2004). The many roles of computation in drug discovery. Science.

[44] Kharkar P.S., Reith M.E., Dutta A.K. (2008). Three-dimensional quantitative structure-activity relationship (3D QSAR) and pharmacophore elucidation of tetrahydropyran derivatives as serotonin and norepinephrine transporter inhibitors. J. Comput. Aided Mol. Des..

[45] Wang L., Xiong L., Wu Z., Miao X., Liu Z., Li D., Zou Q., Yang Z. (2018). Expression of UGP2 and CFL1 expression levels in benign and malignant pancreatic lesions and their clinicopathological significance. World J. Surg. Oncol..

[46] Tan G.S., Lim K.H., Tan H.T., Khoo M.L., Tan S.H., Toh H.C., Ching Ming Chung M. (2014). Novel proteomic biomarker panel for prediction of aggressive metastatic hepatocellular carcinoma relapse in surgically resectable patients. J. Proteome Res..

[47] Li Y., Zhuang H., Zhang X., Li Y., Liu Y., Yi X., Qin G., Wei W., Chen R. (2018). Multiomics Integration Reveals the Landscape of Prometastasis Metabolism in Hepatocellular Carcinoma. Mol. Cell. Proteom..

[48] Tan Z., Chaudhai R., Zhang S. (2016). Polypharmacology in Drug Development: A Minireview of Current Technologies. ChemMedChem.

[49] Saenz-Mendez P., Eriksson L.A. (2018). Exploring Polypharmacology in Drug Design. Methods Mol. Biol..

[50] Rosini M. (2014). Polypharmacology: The rise of multitarget drugs over combination therapies. Future Med. Chem..

[51] Antolin A.A., Workman P., Mestres J., Al-Lazikani B. (2016). Polypharmacology in Precision Oncology: Current Applications and Future Prospects. Curr. Pharm. Des..

[52] Anighoro A., Bajorath J., Rastelli G. (2014). Polypharmacology: Challenges and opportunities in drug discovery. J. Med. Chem..

[53] Reddy A.S., Zhang S. (2013). Polypharmacology: Drug discovery for the future. Expert Rev. Clin. Pharm..

[54] Bolognesi M.L., Cavalli A. (2016). Multitarget Drug Discovery and Polypharmacology. ChemMedChem.

[55] Chen D., Zhang H., Lu P., Liu X., Cao H. (2016). Synergy evaluation by a pathway-pathway interaction network: A new way to predict drug combination. Mol. Biosyst..

[56] Coss K.P., Colin P.H., Barnara A., Stockmamm H., Crushell E., Saldova R., Knerr I., Gozalbo M.E.R., Monavari A., Rudd P.M. (2014). N-Glycan abnormalities in children with Galactosemia. J. Proteome Res..

[57] Liu Y., Xia B., Gleason T.J., Castaneda U., He M., Berry G.T., Fridovich-Keil J.L. (2012). N- and O-linked glycosylation of total plasma glycoproteins in galactosemia. Mol. Genet. Metab..

[58] Maratha A., Colhoun H.O., Knerr I., Coss K.P., Doran P., Treacy E.P. (2016). Classical Galactosaemia and CDG, the N-Glycosylation Interface. A Review. JIMD Rep..

[59] Charlwood J., Clayton P., Keir G., Mian N., Winchester B. (1998). Defective galactosylation of serum transferrin in galactosemia. Glycobiology.

[60] Dobbie J.A., Holton J.B., Clamp J.R. (1990). Defective galactosylation of proteins in cultured skin fibroblasts from galactosaemic patients. Ann. Clin. Biochem..

[61] Ornstein K.S., McGuire E.J., Berry G.T., Roth S., Segal S. (1992). Abnormal galactosylation of complex carbohydrates in cultured fibroblasts from patients with galactose-1-phosphate uridyltransferase deficiency. Pediatric Res..

[62] Sturiale L., Barone R., Fiumara A., Perez M., Zaffanello M. (2005). Hypoglycosylation with increased fucosylation and branching of serum transferrin N-glycans in untreated galactosemia. Glycobiology.

[63] Lai K., Langley S.D., Khwaja F.W., Schmitt E.W., Elsas L.J. (2003). GALT deficiency causes UDP-hexose deficit in human galactosemic cells. Glycobiology.

[64] Ng W.G., Xu Y.K., Kaufman F.R., Donnell G.N., Wolff J., Allen R.J., Koritala S., Reichardt J.K. (1994). Biochemical and molecular studies of 132 patients with galactosemia. Hum. Genet..

[65] Ng W.G., Xu Y.K., Kaufman F.R., Donnell G.N. (1993). Measurements of uridine diphosphate hexoses in galactosemia. J. Pediatr..

[66] Wang B.B., Xu Y.K., Ng W.G., Wong L.J. (1998). Molecular and biochemical basis of galactosemia. Mol. Genet. Metab..

[67] Avendaño C., Menéndez J.C. (2008). Medicinal Chemistry of Anticancer Drugs, 2008.

[68] Jhoti H., Williams G., Rees D.C., Murray C.W. (2013). The ‘rule of three’ for fragment-based drug discovery: Where are we now?. Nat. Rev. Drug Discov..

[69] Lai K., Willis A.C., Elsas L.J. (1999). The biochemical role of glutamine 188 in human galactose-1-phosphate uridyltransferase. J. Biol. Chem..

[70] Lai K., Elsas L.J. (2000). Overexpression of human UDP-glucose pyrophosphorylase rescues galactose-1-phosphate uridyltransferase-deficient yeast. Biochem. Biophys. Res. Commun..

[71] Fuhring J.I., Cramer J.T., Schneider J., Baruch P., Gerardy-Schahn R., Fedorov R. (2015). A quaternary mechanism enables the complex biological functions of octameric human UDP-glucose pyrophosphorylase, a key enzyme in cell metabolism. Sci. Rep..

[72] Peneff C., Ferrari P., Charrier V., Taburet Y., Monnier C., Zamboni V., Winter J., Harnois M., Fassy F., Bourne Y. (2001). Crystal structures of two human pyrophosphorylase isoforms in complexes with UDPGlc(Gal)NAc: Role of the alternatively spliced insert in the enzyme oligomeric assembly and active site architecture. EMBO J..

