# Supplementary material for: Discovery of Novel Inhibitors Targeting Multi-UDP-hexose Pyrophosphorylases as Anticancer Agents
Source: Molecules. 2020 Feb 3;25(3):645. doi: 10.3390/molecules25030645 (PMC7038226; doi:10.3390/molecules25030645)
Supplement: Supplementary file 1 [file molecules-25-00645-s001.pdf]

molecules

Discovery of novel inhibitors targeting multi-UDP-hexose pyrophosphorylases as anticancer agents

Yueqin Yang [1], Hariprasad Vankayalapati [2,3], Manshu Tang [1], Yingbo Zheng [4], Yingri Li [1], Cong Ma [4,*] and Kent Lai [1,*]

1 Department of Pediatrics, University of Utah School of Medicine, Salt Lake City, UT, USA; Yueqin.Yang@hsc.utah.edu (Y.-Q. Y.), manshu.tang@hsc.utah.edu (M.-S. T.), annie826.li@gmail.com (Y.-R. L.)
2 Department of Medicinal Chemistry, University of Utah School of Pharmacy, Salt Lake City, UT, USA; hari@hci.utah.edu
3 Huntsman Cancer Institute, University of Utah, Salt Lake City, UT, USA; hari@hci.utah.edu
4 State Key Laboratory of Chemical Biology and Drug Discovery, Department of Applied Biology and Chemical Technology, Hong Kong Polytechnic University, Kowloon, Hong Kong; jason.zheng@polyu.edu.hk
* Correspondence: cong.ma@polyu.edu.hk (C.M.); kent.lai@hsc.utah.edu (K.L.)

Supplementary Table S1. Nucleotide sequence of primers used for *GALE*, *UGDH* and *AGX1/UAP1* Cloning.

Gene Name	Genbank Accession	Primer	Primer sequence (5′-3′)
GALE	NM_000403.3	Forward	CATTCGATATCTCAGATCTATGGCAGAGAAGGTGCTGG
		Reverse	TGAACCTCGAGCTATCAGGCTTGCGTGCCAA
UGDH	NM_003359.4	Forward	CATTCTGATCAATGTTTGAAATTAAGAAGATCTGTTGC
		Reverse	TGAACCTCGAGCTACTACACTTTAGGTTTCTTGTTAGGT
AGX1/UAP1	NM_001324116.1	Forward	CATTCGGATCCATGAACATTAATGACCTCAAACTCA
		Reverse	TGAACGTCGACCTATCAAATACCATTTTTCACCAGCT

Supplementary Table S2. siRNA nucleotide sequence.

siRNA Name		Sequence (5′-3′)
**siRNA *GALT***	Sense	GGCACUUCCUGAGGUUCAUTT
	Anti-sense	AUGAACCUCAGGAAGUGCCTT
**siRNA *UGP2***	Sense	GAAGGUUCAAGAUUAUCUATT
	Anti-sense	UAGAUAAUCUUGAACCUUCGT
**siRNA *AGX1***	Sense	GCAACAGCUCAAAAACGAATT
	Anti-sense	UUCGUUUUUGAGCUGUUGCCA
**siRNA Control**	Cat. No. AM4613	Life technologies

Supplementary Table S3. Nucleotide sequence of primers used for RT-PCR.

Primer Name	Primer sequence (5′-3′)
**GALT Forward**	AAATGCTTGCTCAGGCTCAGA
**GALT Reverse**	ACCTCAGGAAGTGCCCTTAG
**UGP2 Forward**	CCTTCTGTGGATTGGGGAAA
**UGP2 Reverse**	AGCCCCTGGCCTTTATCTTT
**UAP1 Forward**	CAAGGGATCAAGATCAGCTCCA
**UAP1 Reverse**	CCCTGCCCACCAGCTAGAAG
**GAPDH Forward**	CCATGGGGAAGGTGAAGGTC
**GAPDH Reverse**	AGTTAAAAGCAGCCCTGGTGA

Supplementary Table S4. Enzymatic Activity (25 μM GAL-012) as % of vehicle (DMSO).

	**Vehicle**	**GALT**	**GALE**	**UGP2**	**UGDH**	**AGX1/UAP1**
**GAL-012**	100%	63.20%	100%	58.46%	100%	56.45%
